# Interrater reliability of the mind map assessment rubric in a cohort of medical students

**DOI:** 10.1186/1472-6920-9-19

**Published:** 2009-04-28

**Authors:** Anthony V D'Antoni, Genevieve Pinto Zipp, Valerie G Olson

**Affiliations:** 1 Department of Graduate Programs in Health Sciences, School of Health and Medical Sciences, Seton Hall University, 400 South Orange Avenue, South Orange, NJ 07079, USA; 2 Division of Pre-clinical Sciences, New York College of Podiatric Medicine, 1800 Park Avenue, New York, NY 10035, USA

## Abstract

**Background:**

Learning strategies are thinking tools that students can use to actively acquire information. Examples of learning strategies include mnemonics, charts, and maps. One strategy that may help students master the tsunami of information presented in medical school is the mind map learning strategy. Currently, there is no valid and reliable rubric to grade mind maps and this may contribute to their underutilization in medicine. Because concept maps and mind maps engage learners similarly at a metacognitive level, a valid and reliable concept map assessment scoring system was adapted to form the mind map assessment rubric (MMAR). The MMAR can assess mind map depth based upon concept-links, cross-links, hierarchies, examples, pictures, and colors. The purpose of this study was to examine interrater reliability of the MMAR.

**Methods:**

This exploratory study was conducted at a US medical school as part of a larger investigation on learning strategies. Sixty-six (*N *= 66) first-year medical students were given a 394-word text passage followed by a 30-minute presentation on mind mapping. After the presentation, subjects were again given the text passage and instructed to create mind maps based upon the passage. The mind maps were collected and independently scored using the MMAR by 3 examiners. Interrater reliability was measured using the intraclass correlation coefficient (*ICC*) statistic. Statistics were calculated using SPSS version 12.0 (Chicago, IL).

**Results:**

Analysis of the mind maps revealed the following: concept-links *ICC *= .05 (95% CI, -.42 to .38), cross-links *ICC *= .58 (95% CI, .37 to .73), hierarchies *ICC *= .23 (95% CI, -.15 to .50), examples *ICC *= .53 (95% CI, .29 to .69), pictures *ICC *= .86 (95% CI, .79 to .91), colors *ICC *= .73 (95% CI, .59 to .82), and total score *ICC *= .86 (95% CI, .79 to .91).

**Conclusion:**

The high *ICC *value for total mind map score indicates strong MMAR interrater reliability. Pictures and colors demonstrated moderate to strong interrater reliability. We conclude that the MMAR may be a valid and reliable tool to assess mind maps in medicine. However, further research on the validity and reliability of the MMAR is necessary.

## Background

A learning strategy is a thinking tool that a student can use to actively acquire information and some examples include mnemonics, charts, and maps [1]. In recent years, several papers on the use of teaching and learning strategies in medical education have been published that include case-based teaching [2], web-based teaching [3], didactic learning, and problem-based learning (PBL) [4,5]. These innovative strategies help students learn and ultimately integrate information [1]. Although these learning strategies may differ in efficacy and applicability, they are all rooted in a conceptual framework called the constructivist theory of learning, which states that meaningful learning, or learning with understanding [6], occurs when learners assimilate new information within their existing framework [1,7,8]. Constructivism also underlies two learning strategies that hold promise in medical education–namely, concept mapping and mind mapping.

### Concept Maps and Mind Maps

The concept map strategy, which was developed by Joseph Novak [9], uses hierarchical order to link concepts together with propositions, or the linking of words, between concepts. These propositions indicate units of meaning that highlight the relationship between concepts [10], and the cross-links show relationships between concepts that would otherwise be unrecognized using a non-mapping learning strategy. Because the student creates the concept map without a template, the map ultimately represents the student's own interpretation of ideas.

**Figure 1:**
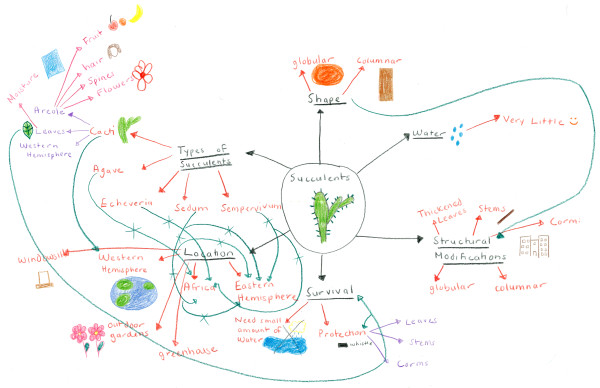
**Student mind map**. An example of a high-scoring mind map from one of the medical students in this study. AVD assigned this mind map a total score of 400, GPZ assigned it a total score of 337, and VGO assigned it a total score of 377. The average total score of this mind map, based on all 3 examiners, was 371.33. Note the hierarchical organization of the mind map and the effective use of pictures and colors. In addition, this map contains numerous cross-links, which resulted in higher scores.

Like concept maps, another mapping strategy that relies on student interpretation and understanding is the mind map strategy. This learning strategy has not been widely used in medical education despite recent research suggesting that mind mapping improves long-term memory in medical students [11]. Mind mapping was developed by Tony Buzan [12] and inspired by the notebooks of Leonardo da Vinci [13]. Unlike most learners' notes, da Vinci's notes were not linear but elliptical–he used pictures and text to illustrate ideas and often connected different concepts on the same page. Mind maps, like da Vinci's notes, are multi-sensory tools that use visuospatial orientation to integrate information, and consequently, help students organize and retain information [14]. An example of a mind map created by a medical student in this study is shown in Figure 1. Other examples of mind maps created by health professional students have been published elsewhere [15,16].

Although the amount of information that medical students are expected to retain is voluminous [17], there are limited learning strategies available to these students to master the information and succeed in medical school [18]. Given this very large amount of information, many medical students resort to passive learning, a phenomenon that has been shown to increase the risk of academic failure because it leads to poor retention [19]. Passive learning refers to learning strategies that emphasize memorization without an attempt to connect and understand information. Passive learners are not stimulated cognitively during the learning process and do not attempt to form connections between information [1]. In contrast, active learning encourages this interconnectivity and engages the learner in activities that promote meaningful learning [1]. Both concept maps and mind maps promote active learning on a metacognitive level and differ only in their organization of information.

The mind map strategy may be a useful tool that medical students can use to facilitate the learning process. However, before mind mapping is used in medical education, there exists a need to develop a valid and reliable rubric that can be used to objectively score mind maps. This rubric should reflect the nature of understanding among the students who have created them, and also be valid and reliable.

### Related Literature

West, Pomeroy, Park, Gerstenberger, and Sandoval [20] studied the validity and reliability of concept mapping assessment (CMA) in graduate medical education. These authors investigated whether concept maps can be scored reliably and whether CMA can measure changes in the conceptual framework of resident physicians. A sample of 21 pediatric resident physicians (*N *= 21) were given a training session on how to construct concept maps and then asked to draw pre-instruction concept maps on the topic of seizures. Subjects then participated in a 3-session seizure education course and were asked to draw post-instruction concept maps. The maps were independently scored by 3 blinded raters and the interrater reliability was measured. The raters underwent a 30-minute concept map scoring training seminar prior to scoring the concept maps. Scores were based upon the following variables: concept links (2 points), level of hierarchy (5 points), cross-links (10 points), and examples (1 point). For each concept map, total scores and subscores for each variable were generated and the correlation between raters' scores and subscores was determined using the Spearman rank correlation statistic. Pre-instruction and post-instruction scores were compared using the Wilcoxon signed rank test.

Mean scores of pre- and post-instruction concept maps increased significantly (*p *= .03). In addition, cross-links (*p *= .02) and concept links (*p *= .01) increased significantly. These results suggested that learning occurred as a result of the 3-session seizure education course. The authors assumed that the physicians' conceptual framework changed based not only on these results, but on the qualitative nature of the post-instruction maps, which were more complex with increased cross-linking than the pre-instruction maps. Interrater correlation of scores was weak to moderate for the pre-instruction maps (*r *= .51–.69), and moderate to strong for the post-instruction maps (*r *= .74–.88). These data suggested that concept maps can be reliably scored and can gauge the level and complexity of knowledge accrued by physicians as they progress through their residencies [20].

In a later study, West, Park, Pomeroy, and Sandoval [21] compared 2 CMA scoring systems (structural and relational methods) using a methodology similar to their previous study [20]. The CMA structural method scoring system assigns weighted numerical scores based upon hierarchical structure, cross-links, and concept-links [9,21]. In contrast, the CMA relational method scoring system is based on the quality of each concept-link, without considering the structure of the map [21]. Interrater correlations were found to be strong with the relational method (*r *= .96 for pre-instructional maps and *r *= .91 for post-instructional maps) and moderate to strong with the structural method (*r *= .65 for pre-instructional maps and *r *= .84 for post-instructional maps) [21]. However, further analysis of the data revealed that the relational method failed to demonstrate the validity seen in the structural method. Therefore, the structural method was more sensitive than the relational method in measuring changes and differences in concept maps [21]. Another study investigating the reliability of CMA was recently published and the interrater reliability (G-coefficient) of the CMA structural scoring system was found to be .96 and .93 for the topics of asthma and diabetes, respectively [22].

In the present study, the CMA structural scoring system was adapted because of the identical nature of how concept maps and mind maps are believed to reach the metacognitive level of the learner. In addition, the CMA structural scoring system has been shown to be valid and reliable in a similar group of subjects (ie, physicians). This adaptation led to the creation of the mind map assessment rubric (MMAR), which was used to assess the quality of student mind maps in the present study. As depicted in Table 1, the MMAR uses the CMA structural scoring system as a framework with the addition of 2 components unique to mind maps–pictures and colors. For each picture drawn, 5 points was assigned to the mind map and for each color used, 5 points was assigned to the mind map.

**Table 1:** Mind map assessment rubric

Weighted scores based on hierarchical structure, cross-links, concept-links, pictures, and color.
**Scoring**

• Concept-links (2 points each)
• Cross-links (10 points each)
• Hierarchies (5 points each)
• Examples (1 point each)
• Invalid components (0 points)
• Pictures (5 points each)
• Colors (5 points each)

*Note*. Adapted from West et al. [21].

The purpose of this preliminary study was to assess the feasibility of using the MMAR to score mind maps in a cohort of medical students and to report its interrater reliability.

## Methods

The design of this study was exploratory and quantitative in nature. The study was conducted at a medical school located in a large metropolitan area in the Northeastern United States. The study was fully approved by an Institutional Review Board prior to the study announcement and subject recruitment.

The study was conducted on a half-day during orientation week. A total of 66 (*N *= 66) matriculated first-year medical students volunteered to participate in the study. Subjects were asked to read and learn information contained in a 394-word text passage. The text passage was adopted from the verbal ability section of a previously published Graduate Record Examination (GRE), and dealt with the topic of cacti and other succulent plants. This text passage was chosen because it was specifically written for undergraduate students who want to pursue graduate training, and therefore, matched the academic level of the medical students in the sample. Most of the medical students had never taken the GRE because entry into a United States medical school requires students to take the Medical College Admissions Test (MCAT) and not the GRE.

After all the subjects entered the lecture hall, they were asked to sit in any seat. At each seat was placed the following: a large, numeric-coded sealed catalog envelope, 2 standard pencils, and one set of 12 colored pencils.

Subjects were asked to open the catalog envelope and remove Packet 1, which contained a demographic survey. Subjects were given 5 minutes to complete the demographic survey using the standard pencils. At the end of this period, subjects were asked to place the demographic survey back into Packet 1, and place Packet 1 back into the catalog envelope.

The subjects then participated in a 30-minute PowerPoint presentation on the topic of mind map usage and construction by an instructor experienced in mind mapping. After the presentation, subjects were asked to open up the catalog envelope and remove Packet 2, which contained the text passage and a numeric-coded blank piece of paper that measured 12 by 18 inches. According to Buzan and Buzan [12], a mind map should be drawn on blank paper that is larger than standard 8 1/2 by 11-inch paper. The rationale behind using larger paper is to allow the student to break away from the boundaries inherent in standard size paper and thus propagate creativity. The use of lined paper is discouraged because it theoretically restricts thought [13]. Subjects were told to place the blank paper in landscape orientation and use the colored pencils to create mind maps based upon the text passage. They were given 25 minutes to create their mind maps. After this period, subjects were asked to place the text passage and mind map back into Packet 2, and place Packet 2 back into the catalog envelope. At the conclusion of the study, all catalog envelopes were collected and a total of 66 mind maps were available for analysis.

Interrater reliability refers to the variations that exist among two or more human examiners [23]. In the present study, interrater reliability was measured among 3 examiners who used the MMAR to score the mind maps. This was accomplished using the intraclass correlation coefficient (*ICC*) as an index to reflect both correlation and agreement among the examiners. The *ICC *range is from 0 to 1 and there are six methods of *ICC *[24]. The second method (covariance matrix) was chosen because the 3 examiners are representative of a larger population of similar examiners [23]. In this method, analysis of variance (ANOVA) is used with the examiner as the independent variable [25].

All statistics reported in this study were calculated using SPSS version 12.0 (Chicago, IL) at an α = .05.

## Results

### Demographics of Subjects

The sample consisted of 31 males (47%) and 35 females (53%). The ethnicities of these subjects were 3 African Americans (4.7%), 35 Caucasians (54.7%), 18 Asian Americans/Pacific Islanders (28.1%), 3 Hispanics/Latinos/Mexican Americans (4.7%), and 5 Mixed/Other (7.8%). These data are depicted in Table 2. The mean age of subjects was 24.74 years (*SD *= 3.91). Subjects had a mean total SAT score of 1254.46 (*SD *= 110.20) and their SAT subscores are reported in Table 3. The mean total MCAT score of subjects was 27.05 (*SD *= 3.17). MCAT biology, physics, and verbal subscores are also reported in Table 3.

**Table 2:** Demographics of subjects

		Subjects (*N *= 66)
Gender	Male	31 (47.0%)^a^
	Female	35 (53.0%)
		
		Subjects (*N *= 64)^b^
Ethnicity	African American	3 (4.7%)
	Anglo American, Caucasian	35 (54.7%)
	Asian American/Pacific Islander	18 (28.1%)
	Hispanic, Latino, Mexican American	3 (4.7%)
	Mixed/Other	5 (7.8%)

^a ^Data are presented as number of subjects (percentage).

^b ^Two subjects did not disclose ethnicity.

**Table 3:** SAT and MCAT scores of subjects

Variable	*M*	*N*^a^	*SD*
Subjects (*N *= 66)			
Age	24.74	66	3.91
SAT (Total)	1254.46	56	110.20
SAT (Verbal)	623.08	39	65.58
SAT (Math)	654.10	39	66.44
MCAT (Total)	27.05	66	3.17
MCAT (Biology)	9.52	62	1.30
MCAT (Physics)	9.02	62	1.54
MCAT (Verbal)	8.68	62	1.80

^a ^For some variables, *N *changed because subjects did not recall or never took the assessment (ie, some students took the ACT instead of the SAT).

*Note*. ACT (American College Test); SAT (Scholastic Aptitude Test); Medical College Admissions Test (MCAT).

### Descriptive Statistics of Mind Map Scores

Mind map quality was assessed using the MMAR scoring grid (see Additional file 1). When using the MMAR, an examiner assigns a total numeric score based on several variables (see Table 1). In this study, 3 examiners (AVD, GPZ, and VGO) scored the mind maps of all subjects and verified the face validity of the MMAR. The examiners were experienced medical educators who had used mind maps in the academic setting and participated in previous mind map research. After the study was completed, the examiners scored all 66 mind maps independently.

Descriptive statistics of the mind map scores between the examiners are found in Table 4. Examiner 1 (AVD) recorded the following for total mind map score of all 66 mind maps: *M *= 200 and *SD *= 55.50 with a *Min *score of 102 and a *Max *score of 400. Examiner 2 (GPZ) recorded the following for total mind map score of all 66 mind maps: *M *= 175.47 and *SD *= 63.22 with a *Min *score of 92 and a *Max *score of 415. Examiner 3 (VGO) recorded the following for total mind map score of all 66 mind maps: *M *= 279.35 and *SD *= 77.77 with a *Min *score of 134 and a *Max *score of 539. Data for separate variables are found in Table 4.

**Table 4:** Descriptive statistics of mind map scores between three examiners

Variable	*Min*	*Max*	*M*	*SD*
Examiner 1 (AVD)				
Concept-links	4	106	38.97	20.43
Cross-links	0	130	23.03	25.05
Hierarchies	10	25	17.88	3.72
Examples	4	31	15.65	5.75
Pictures	5	135	59.39	27.63
Colors	20	60	45.08	10.72
Total score	102	400	200.00	55.50

Examiner 2 (GPZ)				
Concept-links	0	10	1.12	2.22
Cross-links	0	200	35.91	41.98
Hierarchies	0	105	50.53	20.51
Examples	2	19	8.44	3.86
Pictures	0	120	46.52	25.41
Colors	20	45	32.95	5.94
Total score	92	415	175.47	63.22

Examiner 3 (VGO)				
Concept-links	0	16	4.48	3.93
Cross-links	0	300	53.48	58.05
Hierarchies	5	350	117.80	62.95
Examples	0	53	20.55	11.28
Pictures	0	105	48.71	29.10
Colors	20	55	34.32	8.54
Total score	134	539	279.35	77.77

### Interrater Reliability of the MMAR

The results of the *ICC *analysis for 66 mind map scores based on 3 examiners follow: concept-links *ICC *= .05 (95% CI, -.42 to .38), cross-links *ICC *= .58 (95% CI, .37 to .73), hierarchies *ICC *= .23 (95% CI, -.15 to .50), examples *ICC *= .53 (95% CI, .29 to .69), pictures *ICC *= .86 (95% CI, .79 to .91), colors *ICC *= .73 (95% CI, .59 to .82), and total score *ICC *= .86 (95% CI, .79 to .91). These data are also found in Table 5. The high *ICC *values for pictures and colors suggest that these 2 constructs can be reliably measured using the MMAR. Although the *ICC *values for cross-links and examples were significant, they were only moderately reliable. The strong *ICC *value for total score suggests that the MMAR can be reliably scored among examiners.

**Table 5:** Intraclass correlation coefficients of mind map scores of three examiners

Variable	*ICC*	*95% CI*
Concept-links	.05	-.42 to .38
Cross-links *	.58	.37 to .73
Hierarchies	.23	-.15 to .50
Examples *	.53	.29 to .69
Pictures *	.86	.79 to .91
Colors *	.73	.59 to .82

Total score *	.86	.79 to .91

*Note*. Significant differences were tested at the 95% confidence interval.

*p < .05.

Internal consistency analyses using Cronbach α were performed on the entire MMAR (all 6 variables) and with the variables pictures and colors excluded. Initial results indicated that the novel variables of the MMAR–pictures and colors–did not strongly strengthen the overall reliability of the MMAR. Cronbach α was found to be .38 (95% CI, .11 to .58) for all 6 variables of the MMAR, and .29 (95% CI, -.02 to .53) when pictures and colors were excluded. In order to get a better understanding of the interrelations of the variables in the rubric, therefore, we ran a principal components analysis on the rubric that included all the variables. As a result of the analysis, two principal factors (ie, sets of variables) were found to be closely related. Factor 1 included the variables cross-links, examples, and hierarchy. Factor 2 included the variables colors, pictures, and cross-links. Consequently, reliabilities on Factors 1 and 2 were performed based on the results of the principal components analysis and yielded the following: Cronbach α for Factor 1 was .57 (95% CI, .35 to .72), and Cronbach α for Factor 2 was .39 (95% CI, .08 to .60).

## Discussion

Figure 1 is an example of a high-scoring mind map from one of the subjects. AVD assigned this mind map a total score of 400, GPZ assigned it a total score of 337, and VGO assigned it a total score of 377. The average total score of this mind map, based on all 3 examiners, was 371.33. This mind map will be used as an example in the following discussion.

The MMAR contains 6 variables (concept-links, cross-links, hierarchies, examples, pictures, and colors) that assess the quality of a mind map and translates this into a numeric score that can enable an educator to compare one mind map from another.

The operational definitions of 4 variables were adapted from the work of West, Pomeroy, Park, Gerstenberger, and Sandoval [20,21]. A concept is a perceived regularity in events or objects designated by a label [21]. As can be observed in Figure 1, there are many concepts in this mind map such as *succulents *and *structural modifications*. A concept-link is a valid link between concepts using a line with a word or statement written above the line describing how the concepts are related [21]. On the right side of the mind map, there is a concept-link between the central theme of the mind map, *succulents and cacti *(note how the subject did not write the word "cactus" but simply represented it as a picture), and *structural modifications*. This is a valid link because the text passage described the different structural modifications found in cacti and other succulent plants. A cross-link is a valid link demonstrating a relationship between different domains of knowledge [21]. Cross-links are very important in mind maps because they demonstrate relationships between concepts. On the left side of the mind map, there are several blue cross-links between different *types of succulents *and *Africa*. Hierarchy is indicated by the direction of the line in the concept-link and the arrangement of concepts in the mind map–that is, more general concepts are located centrally and more specific concepts are located peripherally [21]. In the example, the link from *succulents and cacti *to *structural modifications *to *thickened leaves *represents secondary hierarchy that is valid and unidirectional. An example is a valid word that exemplifies the concept [21]. In Figure 1, the words written in purple–such as *leaves*, *stems*, and *corms*–are examples.

The final 2 variables unique to mind maps that were included in the MMAR are pictures and colors, both of which facilitate the conversion of information from short- to long-term memory [26]. A picture is a graphic representation that aids the learner in recalling the information [15]. Pictures can be located anywhere in the mind map and the combination of the two cortical skills of words and pictures enhances intellectual power [12]. The quality, clarity, and detail of the pictures were not a factor in assigning points. In Figure 1, the subject integrated many pictures into the mind map. Each color used in the mind map was assigned points so that the more colors used, the more points assigned to the mind map. Again, in Figure 1 the subject used many colors when creating the mind map. The advantage of using different colors in a mind map is that it improves the recall of information, allows faster access to the information, and ultimately increases creativity [12].

In this study, we have demonstrated a high *ICC *total score value (.86) among 3 examiners using the MMAR. This finding suggests that the MMAR can be reliably used to score mind maps and demonstrates its potential applications in research and education. Moreover, the addition of pictures and colors reveals strong interrater reliability (.86 and .73, respectively). This suggests that these variables can also be reliably scored and lend to the uniqueness of mind maps. However, pictures and colors were not found to strongly strengthen the overall reliability of the MMAR based on our internal consistency analyses using Cronbach α. Recall Cronbach α was .38 (95% CI, .11 to .58) for all 6 variables of the MMAR and .29 (95% CI, -.02 to .53) without pictures and colors. Cross-links (.58) and examples (.53) were moderately reliable. In this study, concept-links and hierarchies were found to have very weak reliability, and we believe that this was due to confusion as to their differences. Each cross-link in a mind map should be assigned a numeric value (10 points each). Unlike cross-links, however, only the highest level of hierarchy is scored on a mind map (5 points each). For example, the mind map in Figure 1 has quaternary (fourth-level) hierarchy, which can be observed in the left upper quadrant of the mind map. Ultimately, there should be 20 points assigned for hierarchy in this mind map. Cronbach α for Factor 1, with the variable hierarchy excluded (ie, only cross-links and examples), was .75. This suggests that hierarchy is difficult to assess in mind maps, and that further development of this variable is necessary in order to improve its reliability. Further research should explore how different operational definitions for concept-links and hierarchy impact the validity and reliability of the MMAR.

In this study, we did not attempt to create an original mind map grading rubric. Rather, we used a valid and reliable CMA structural scoring system specifically studied in resident physicians as a framework for the MMAR [20,21]. The metacognitive similarities between concept maps and mind maps allowed us to use the CMA structural scoring system as the foundation for the MMAR. Although we have established the interrater reliability of the MMAR, further research is needed to investigate its construct validity and reliability before it can be used in medical education.

## Conclusion

Based upon our findings, we suggest that the MMAR may be a valid and reliable tool that can be used to detect changes in knowledge among medical students. However, additional validity and reliability studies of the MMAR should be conducted to further substantiate our findings.

## Competing interests

The authors declare that they have no competing interests.

## Authors' contributions

AVD conceived the design of the study, scored the mind maps, analyzed the data, and drafted the manuscript. GPV participated in the design of the study, scored the mind maps, and drafted the manuscript. VGO participated in the design of the study, scored the mind maps, and helped draft the manuscript. All authors read and approved the final manuscript.

## Pre-publication history

The pre-publication history for this paper can be accessed here:



## Supplementary Material

Additional File 1 **Mind map assessment rubric**. The information provided includes the operational definitions of the MMAR variables and the actual MMAR scoring grid, which can be used to grade mind maps. Click here for file

## References

[B1] Gage NL, Berliner DC (1998). Educational psychology.

[B2] Kim S, Phillips WR, Pinsky L, Brock D, Phillips K, Keary J (2006). A conceptual framework for developing teaching cases: A review and synthesis of the literature across disciplines. Medical Education.

[B3] Zajaczek JE, Gotz F, Kupka T, Behrends M, Haubitz B, Donnerstag F, Rodt T, Walter GF, Matthies HK, Becker H (2006). eLearning in education and advanced training in neuroradiology: Introduction of a web-based teaching and learning application. Neuroradiology.

[B4] Barrows HS (1994). Practice-based learning: Problem-based learning applied to medical education.

[B5] Dolmans DH, De Grave W, Wolfhagen IH, Vleuten CP van der (2005). Problem-based learning: Future challenges for educational practice and research. Medical Education.

[B6] Daley BJ, Shaw CR, Balistrieri T, Glasenapp K, Piacentine L (1999). Concept maps: A strategy to teach and evaluate critical thinking. Journal of Nursing Education.

[B7] Ausubel DP (1978). Educational psychology: A cognitive view.

[B8] Bodner GM (1986). Constructivism: A theory of knowledge. Journal of Chemical Education.

[B9] Novak JD, Gowin DB (1984). Learning how to learn.

[B10] Irvine LM (1995). Can concept mapping be used to promote meaningful learning in nurse education?. Journal of Advanced Nursing.

[B11] Farrand P, Hussain F, Hennessy E (2002). The efficacy of the 'mind map' study technique. Medical Education.

[B12] Buzan T, Buzan B (1993). The mind map book.

[B13] Gelb MJ (1998). How to think like Leonardo da Vinci: Seven steps to genius every day.

[B14] McDermott P, Clarke DN (1998). Mind maps in medicine.

[B15] D'Antoni AV, Pinto Zipp G (2006). Applications of the mind map learning technique in chiropractic education: A pilot study and literature review. Journal of Chiropractic Humanities.

[B16] Pinto Zipp G, Maher C, D'Antoni AV (2009). Mind maps: Useful schematic tool for organizing and integrating concepts of complex patient care in the clinic and classroom. Journal of College Teaching and Learning.

[B17] Anderson J, Graham A (1980). A problem in medical education: Is there an information overload?. Medical Education.

[B18] Rye PD, Wallace J, Bidgood P (1993). Instructions in learning skills: An integrated approach. Medical Education.

[B19] Dolan S, Mallott DB, Emery JA (2002). Passive learning: A marker for the academically at risk. Medical Teacher.

[B20] West DC, Pomeroy JR, Park JK, Gerstenberger EA, Sandoval J (2000). Critical thinking in graduate medical education: A role for concept mapping assessment?. Journal of the American Medical Association.

[B21] West DC, Park JK, Pomeroy JR, Sandoval J (2002). Concept mapping assessment in medical education: A comparison of two scoring systems. Medical Education.

[B22] Srinivasan M, McElvany M, Shay JM, Shavelson RJ, West DC (2008). Measuring knowledge structure: Reliability of concept mapping assessment in medical education. Academic Medicine.

[B23] Portney L, Watkins MP (2000). Foundations of clinical research: Applications to practice.

[B24] Shrout PE, Fleiss JL (1979). Intraclass correlations: Uses in assessing reliability. Psychological Bulletin.

[B25] McGraw KO, Wong SP (1996). Forming inferences about some intraclass correlation coefficients. Psychological Methods.

[B26] Day JC, Bellezza FS (1983). The relation between visual imagery mediators and recall. Memory & Cognition.

