# Supplementary material for: Interrater reliability of the mind map assessment rubric in a cohort of medical students
Source: BMC Med Educ. 2009 Apr 28;9:19. doi: 10.1186/1472-6920-9-19 (PMC2683832; doi:10.1186/1472-6920-9-19)
Supplement: Additional File 1 — Mind map assessment rubric. The information provided includes the operational definitions of the MMAR variables and the actual MMAR scoring grid, which can be used to grade mind maps. [file 1472-6920-9-19-S1.doc]

MIND MAP ASSESSMENT RUBRIC

**INSTRUCTIONS:** Please use the rubric on the next page to grade the mind maps. Thank you.

| The grading rubric below assigns weighted scores to the mind maps based on hierarchical structure, cross-links, concept-links, pictures, and color. | |
| --- | --- |
| **Variable** | **Points** |
| • Concept-links  • Cross-links  • Hierarchies  • Examples  • Invalid components  • Pictures  • Colors | • 2 points each  • 10 points each  • 5 points each  • 1 point each  • 0 points  • 5 points each  • 5 points each |

*Operational Definitions:*

**Concept:**  A perceived regularity in events or objects designated by a label.

**Concept-link:**  A valid link between concepts using a line with a word or statement written above the line describing how the concepts are related.

**Cross-link:**  A valid link demonstrating a relationship between different domains of knowledge.

**Hierarchy:** Indicated by the direction of the line in the concept-link and the arrangement of concepts in the mind map (ie, more general concepts located centrally and more specific concepts located peripherally).

**Example:** Usually located towards the end of a concept-link, an example is a valid word or words that exemplifies the concept.

**Picture:** A graphic representation that aids the learner in recalling the information. Pictures can be located anywhere in the mind map. Pictures are to be given points based upon their inclusion in the mind map. The quality, clarity, and detail of the pictures should **not** be a factor in assigning points. That is, all pictures will be assigned points irrespective of their quality, clarity, and detail.

**Color:** Each color used in the mind map will be given points so that the more colors used, the more points assigned to the mind map.

MIND MAP ASSESSMENT RUBRIC

**Scorer Initials:**

Mind Map Identification Number:

| **Mind Map Variable** | **Point Distribution** | |
| --- | --- | --- |
|  | **Number** | **Individual Total** |
| • Concept-links (2 points each) | _______ x 2 = |  |
| • Cross-links (10 points each) | _______ x 10 = |  |
| • Hierarchies (5 points each) | _______ x 5 = |  |
| • Examples (1 point each) | _______ x 1 = |  |
| • Invalid components (0 points) |  |  |
| • Pictures (5 points each) | _______ x 5 = |  |
| • Colors (5 points each) | _______ x 5 = |  |
|  | | |
| **CUMULATIVE TOTAL** | |  |
